# Acute Hemolytic Transfusion Reaction in Group B Recipient Associated with Group A Apheresis Platelet Donor: Case Report and Literature Review

**DOI:** 10.1155/2018/8259531

**Published:** 2018-06-24

**Authors:** Tracy R. Shachner, Christopher T. Clark

**Affiliations:** Department of Pathology, University of Tennessee Graduate School of Medicine, Knoxville, TN 37920, USA

## Abstract

Acute hemolytic transfusion reaction is a known but rare potential adverse event related to platelet transfusion. Most reported cases of platelet-related hemolytic transfusion reaction have resulted from transfusion of platelets from group O donor to group A recipient. We identified only one prior case report in the literature of hemolytic transfusion reactions resulting from transfusion of apheresis platelets from group A donor to group B recipient. In that case report, two platelet units were obtained from a single donation and transfused into two separate patients. Both patients exhibited acute hemolytic reactions. The donor is reported to have high anti-B titers, as well as report of probiotic use. We report a case of acute hemolytic reaction in group B recipient following transfusion of apheresis platelets from group A donor with high-titer anti-B but unknown status of probiotic use. This case demonstrates that while low, there still exists potential risk for hemolysis from out-of-group A plasma transfusion.

## 1. Introduction

There is currently no requirement for ABO compatibility for transfusion of platelet units in the United States (US). The AABB (formerly known as the American Association of Blood Banks) only requires transfusion services to have a policy regarding out-of-group components containing significant amount of incompatible ABO antibodies [1]. Approximately 10% to 40% of platelet transfusions in the US are reportedly ABO-incompatible [2]. There appears to be no consensus on a specific method to minimize transfusion of ABO-incompatible plasma contained within platelet units [3–7]. A retrospective analysis revealed an unspecified adverse event in 3 out of 59,933 out-of-group (ABO-incompatible) platelet transfusions within the Medicare database, suggesting an overall low risk with out-of-group platelet transfusion [8]. Prior reports of acute hemolysis related to platelet transfusion have been primarily when group O platelet unit is transfused into group A recipient [7, 9–11]. Association of donor group A apheresis platelet unit to group B recipient has been reported from a single donor who donated two platelet units in a single donation, transfused to two separate patients, and both patient recipients demonstrating acute hemolysis. The authors report the donor to have high anti-B titers (16,384 at both room temperature {saline} and IgG phases) in addition to recent probiotic use [12]. We report a rare case of acute hemolysis in a group B, Rh(D)-positive patient after receiving a single unit of apheresis platelets from a group A, Rh(D)-positive donor with unknown history of probiotic use.

## 2. Case Presentation

A 53-year-old, blood group B, Rh(D)-positive female with history of metastatic adenocarcinoma status after chemotherapy treatment presented to the emergency department with abdominal pain, vomiting, and fever. She was admitted for a possible small bowel obstruction and sepsis. Upon her admission, her complete blood count (CBC) revealed a normal platelet count of 186 × 10^3^ (reference range: 130–400 × 10^3^/mm^3^), low white blood cell count of 2.7 × 10^3^ cells/mm^3^ (reference range: 4.4–11 × 10^3^ cells/mm^3^), normal hemoglobin level of 15.9 g/dL (reference range: 12–16 g/dL), and a normal hematocrit of 46.6% (reference range: 37–47%). Red blood cell antibody screen was negative. Over the next few days, the patient's platelet count continually decreased, reaching a critical low level of 10 × 10^3^ cells/mm^3^ one week after admission. She received a single unit of apheresis platelets from a group A, Rh(D)-positive donor. The unit initially contained 3.7 × 10^11^ platelets per milliliter (mL) within total volume of 270 mL. The platelet product was suspended in Anticoagulant Citrate Dextrose Solution, Solution A (ACD-A) and transfused on storage day 5. The red blood cell visual count was reported negative. Approximately ten minutes after the transfusion was started, the patient began to complain of severe lower back pain. The pain was described as 10/10, sharp and stabbing. No other signs or symptoms were reported, including no fever or blood pressure changes. The patient received 135 mL (50%) of the platelet product. The primary provider care team was notified, and the patient observed while receiving normal saline at 100 mL/hour. No other treatment was initiated. The patient reports the pain 1 1/2 hours later to be 5/10 and “much better” 2 hours after transfusion. A transfusion reaction work-up was initiated, which revealed a posttransfusion blood sample with visible hemolysis and 1+ positive direct antiglobulin test (C3b and C3d positive; IgG negative). No eluate was performed due to absence of detectable IgG on the posttransfusion DAT sample. Pretransfusion blood sample showed no hemolysis, and pretransfusion DAT was negative. Subsequent Gram stain of the platelet unit revealed no organisms seen. Aerobic and anaerobic cultures of the platelet unit after transfusion showed no growth at 5 days. Initial cultures performed by the blood center also reported no growth at 5 days. No medications or fluids were infused with the product. The results of the work-up were consistent with an acute hemolytic transfusion reaction related to an apheresis platelet unit from a group A, Rh(D)-positive donor to a group B, Rh(D)-positive recipient. The antibodies (anti-B) from the platelet unit were titered and found to be high-titer at 512 (Table 1). Titer performed following the College of American Pathologists (CAP) tube method “uniform procedure” utilizing 0.9% normal saline (NaCl). The hemoglobin 14 hours prior to transfusion was 8.9 g/dL. The hemoglobin 1 hour 15 minutes after transfusion was 7.4 g/dL. From a transfusion medicine standpoint, the patient remained stable throughout the remainder of her hospital admission. The platelet product was donated by an 18-year-old male and was part of a double platelet unit donation. The companion platelet aliquot was transfused at another institution to a group compatible recipient without reported adverse event. The donor had previously donated a double red cell product with no known associated adverse event. Multiple attempts to contact donor after reaction did not receive a response. History of probiotic use was not reported by the donor or asked at time of donation. Status of probiotic use could not be confirmed after donation.

## 3. Discussion

Donor antibodies against human leukocyte antigens (HLA) are a major association reported with transfusion-associated acute lung injury (TRALI) [13]. Group AB plasma is considered the universal donor plasma product and is transfused in emergent situations when the blood type of the recipient is unknown. Prior to 2014, male-only plasma was common TRALI risk-reduction strategy. However, use of female-derived AB plasma was still commonly utilized in emergent situations due to low frequency of AB blood type in the general population and inability to meet the demand for group AB plasma products for emergent use. A 2014 AABB Bulletin reports that postmitigation TRALI risk was 1.8 per million units distributed (99% male donors) for donor plasma blood groups A, B, and O. However, the pre- and postmitigation TRALI risk was essentially unchanged for those receiving group AB plasma (26.3 cases per million units distributed with 60% male donors and 40% female donors). The bulletin states that 82% of plasma-mediated TRALI reported to the American Red Cross (23 cases from 2008 to 2011) involved female donors. As a result, the 2014 AABB Bulletin states that plasma and whole blood from allogeneic donors shall be from males and females who have not been pregnant or from previously pregnant females who have negative test results for HLA antibodies [14]. This resulted in potential significant loss of available AB plasma products, and therefore, the need to transition to emergent use of group A plasma. Transfusion of group A plasma is ABO-compatible for groups A and O recipients. However, transfusion of group A plasma is ABO-incompatible for patients of blood groups AB and B who in turn have potential risk of reaction due to minor incompatibility. Blood groups O and A are the most common blood types. Blood group AB is approximately 4% of the general population. Group B is approximately 9% of Caucasian, 20% of African American, and 25% of Asian populations [15]. Therefore, use of emergent group A plasma will be ABO-compatible for the majority of the general population and ABO-incompatible in the minority. Use of emergent A plasma is thought to have low risk of hemolysis [3, 16–19]. Although one unit of standard apheresis platelets stored in human plasma contains more than one volume equivalent of a standard unit of plasma, there currently is no requirement regarding ABO compatibility for platelet transfusion in adults due to short shelf life of platelet products and availability constraints [1]. While it is important to attempt to limit the amount of out-of-group plasma patients receive, out-of-group platelet transfusions occur every day in the US without report of hemolytic reactions [2]. This is supportive evidence that emergent use of group A plasma is likely to have low risk. In addition, during hemorrhagic massive transfusion events, the patient is also receiving group O red blood cells, likely decreasing the relative risk of hemolysis associated with the group A plasma. There is differing practice regarding titering of out-of-group apheresis platelets and group A plasma units for emergent use. There is also great variation in what is considered low- and high-titer [5, 6, 16, 17, 20, 21]. In our case, the donor had anti-B titer of 512 which is above the cutoff for high titer in our laboratory. Since the majority of reports of acute hemolysis related to platelet transfusion are associated with anti-A from group O apheresis platelets [7, 9–11], our practice is to perform a modified titer for anti-A (isohemagglutinins) of group O apheresis platelet units prior to out-of-group transfusion. We perform a modified titer for anti-A at 250 and if negative for agglutination, the unit is considered low-titer and is available for transfusion to any blood group recipient. If the platelet unit is positive at titer of 250, the unit is designated as high-titer and reserved only for group O recipients. The modified titer is a tube test method in which 996 microliters of 0.9% normal saline (NaCl) is placed in a test tube along with 4 microliters of donor plasma from the platelet unit. In addition, separate tubes for positive control (996 microliters saline, 4 microliters anti-A) and negative control (996 microliters saline, 4 microliters anti-B) are made. One drop of 1 : 250 dilution of each sample is transferred into separate test tubes. One drop of type A reagent red blood cells is added to each tube. After 10 minutes incubation at room temperature, the tubes are spun at 3400 rpm for 15 seconds and read for agglutination. When possible, we transfuse ABO-identical apheresis platelet units to our patients in the neonatal intensive care unit. In addition to screening donors for high-titer ABO antibodies, other suggested strategies to reduce risk of out-of-group platelet transfusion include transfuse ABO-identical platelet units when possible particularly in high-risk patients (neonates, pediatric patients, hematopoietic stem cell transplant, and organ transplant recipients), wash and resuspend platelets in saline, volume reduction, reduce plasma volume of group O apheresis platelet concentrates to 50 mL, and replace plasma with additive solutions or AB plasma after washing [22–29]. We utilize group A plasma in emergent situations, but do not titer or set out-of-group A plasma volume limits. The STAT Study reports that the majority of Level 1 trauma center survey respondents maintain group A plasma for immediate available use and 63% use group A plasma during initial resuscitation phase of trauma patients with unknown ABO group. This study also reports that 62% of respondents do not set limits for potential out-of-group A plasma in trauma patients and only 21% consider anti-B titer in selection of group A plasma units dedicated for emergent use [19, 21].

## 4. Conclusion

There is currently no standard practice for performing titers on emergent group A plasma or for out-of-group apheresis platelet products prior to transfusion. While out-of-group apheresis platelet transfusion is acceptable common practice and emergent use of group A plasma is becoming more common, our case report demonstrates that while low, there still exists potential risk of acute hemolytic transfusion reaction from group A plasma transfused to a group B recipient. It would be helpful to have additional well-controlled studies to evaluate the level of significant isohemagglutinin titers for out-of-group transfusion and recommendation guidelines for standardized practice. Additional investigation into the potential significance of probiotic use in the donor population could also be beneficial.

## Figures and Tables

**Table 1:** Tube method (saline) titer of donor platelet unit with agglutinin reaction strength at room temperature and at 37°C.

Titer	Room temperature at 30 minutes	37°C (IgG) at 30 minutes
1	4+	4+
2	4+	4+
4	4+	4+
8	4+	4+
16	4+	4+
32	4+	4+
64	4+	3+
128	4+	3+
256	3+	2+
512	1+	2+
1024	Negative	Negative
2048	Negative	Negative
4056	Negative	Negative
